# Plasma GDF15 concentration predicts early recurrence after atrial arrhythmia ablation

**DOI:** 10.1172/jci.insight.198444

**Published:** 2026-02-24

**Authors:** Johanna Tennigkeit, Maurice Wiegelmann, Chiara Massa, Jonas Lübcke, Werner Dammermann, Karina Börner, Filip Schröter, Barbara Seliger, Maximilian Kleinert, Oliver Ritter, Gregor Sachse

**Affiliations:** 1 Brandenburg Medical School (Theodor Fontane), ZTM-BB, Brandenburg/Havel, Germany.; 2 University Hospital Brandenburg/Havel, Germany.; 3 Faculty of Health Sciences Brandenburg, Germany.; 4 Heart Center Brandenburg, Bernau, Germany.; 5 Martin-Luther-University Halle, Institute for Pathology, Germany.; 6 Department of Molecular Physiology of Exercise and Nutrition, German Institute of Human Nutrition (DIfE), Potsdam-Rehbruecke, Germany.; 7 Institute of Nutrition Science, University of Potsdam, Germany.

**Keywords:** Cardiology, Immunology, Arrhythmias, Biomarkers, Cellular immune response

## Abstract

<div> <div> <div> <div> <div> <div> A blood marker called GDF15, measured before ablation, can predict arrhythmia recurrence within 24 hours, suggesting immune reactions influence healing and treatment success. </div> </div> </div> </div> </div> </div>

Catheter-based ablation is the first-line therapeutic approach for atrial arrhythmias, a group of prevalent cardiac disorders that are associated with substantial morbidity and diminished quality of life. Scar formation after ablation treatment is crucial for therapeutic success, with recurrence rates correlated to the size of gaps in the scar line (1). Despite advances in ablation technology and methodology, recurrence rates remain high, and it is currently unclear why scar formation is negatively affected in some patient groups, like patients with high BMI (2). Knowledge about the initial inflammatory phase of scar formation is especially lacking, when edema and tissue response can confound the assessment of successful intraprocedural conduction block (3).

One hypothesis is that the immunological state before ablation influences the initial stage of scar formation when soluble factors and cardiac resident immune cells act as initiators of inflammation (4). While resident immune cell state and paracrine activity are difficult to monitor, preablation immunological status can be partially characterized via levels of cytokines and other immunomodulatory molecules. We therefore screened for biomolecules linked to early scar formation by using arrhythmia recurrence at 24 hours as the measurable outcome, when scar formation is dominated by the transient inflammatory response (3). Our study included 72 patients undergoing electrophysiological examination (EP) and catheter ablation for atrial arrhythmia. Participants (45% female) had no cancer, immunosuppressive treatment, or signs of infection. For baseline characteristics, see Supplemental Tables 1 and 2 (supplemental material available online with this article; https://doi.org/10.1172/jci.insight.198444DS1).

For detection in prior-ablation plasma samples, 21 molecular markers were chosen based on association with wound healing or with resident cardiac immune cells (Figure 1A). Biomolecule concentrations and clinically relevant parameters (CRP, NT-proBNP, leukocytes) were then grouped by early recurrence (ER): 32% of patients had experienced early recurrent events, assessed by 12-lead ECG within 24 hours after ablation (ER, *n* = 20, 68% nonearly recurrence [NER], *n* = 43).

Out of the biomolecule candidates, GDF15 levels showed the strongest link to risk of ER (ER: 2.5 ± 1.1 ng/mL, NER: 1.0 ± 0.4 ng/mL, *P* < 0.0001; Figure 1A). Heart failure biomarker NT-proBNP also showed a significant correlation to ER. However, GDF15 levels, unlike NT-proBNP, allowed for separation of ER and NER instances with very little overlap (Figure 1B). This was reflected in receiver operating characteristic (ROC) analysis (Figure 1C), where GDF15 (AUC = 0.98) decisively outperformed NT-proBNP (AUC = 0.8), with a Youden’s index–derived cut-off value of 1.61 ng/mL. Patient characteristics below and above the cut-off value were near identical to the NER and ER group, respectively (Supplemental Table 1).

To exclude a potential exaggeration of GDF15’s predictive power due to correlation with age, sex, BMI, HbA1c, NT-proBNP, or CRP, we performed binomial logistic regression analysis. Regression revealed GDF15 as the only significant predictor out of all parameters modeled (Supplemental Table 3).

Although sample size was insufficient to compare subpathologies, GDF15 remained the best predictor of 24-hour recurrence when restricting the analysis to de novo patients with AF, both in comparison with other cytokines (Supplemental Figure 1) and in ROC analysis compared with NT-proBNP (Figure 1D). There was no association between preprocedural GDF15 levels and paroxysmal versus persistent AF.

## Discussion

Our screening revealed circulating GDF15 concentration prior to ablation as a powerful statistical predictor of 24-hour arrhythmia recurrence. This contrasts with previous studies on atrial fibrillation recurrence at 1 to 24 months after ablation, in which GDF15 had small (5) or no predictive value (Supplemental Material). To our knowledge, this is the first study focusing on GDF15 in the context of 24-hour recurrence specifically, and its findings will need to be replicated in an independent patient cohort.

GDF15 is a member of the TGF-β superfamily that can act as a cytokine and chemokine inhibitor but also regulates appetite, has BMI lowering effects, and is compensatory high in patients with obesity and obese animal models (6). Its low expression in healthy human cardiovascular tissues increases in response to cardiovascular disease, The cell types involved are not known, but might include heart-resident macrophages (6). In our cohort, GDF15 concentrations correlated positively with heart rate, but if this implies a mechanistic link is presently unclear (Supplemental Figure 1).

The role of GDF15 in edema has yet to be studied — now of high interest considering our findings. More is known about GDF15 antiinflammatory function in cardiac tissue. The inflammation processes triggered by cardiac tissue trauma after ablation require a delicate balance between too little and too much recruitment and expansion of immune cells (4). Loss of GDF15 has been shown to cause immune cell overrecruitment after myocardial infarction, resulting in tissue weakening and cardiac rupture (6). Also, GDF15 may acutely influence cardiac wound size independently of recruited immune cells (6). Mechanistically, increased GDF15 levels may influence edema formation or suppress inflammatory cell recruitment after ablation trauma and subsequently suppress stable scar formation, explaining ER of arrhythmias despite previous confirmation of conduction block in the ablation area. After replication of results in an independent cohort, it will be of high interest to investigate the role of GDF15 in ablation edema and scar formation with preclinical models, to determine if high GDF15 levels define a subgroup of patients prone to immediate ER of arrhythmia or if GDF15 itself promotes arrhythmia recurrence through effects on immune cell recruitment, local swelling, cell viability, or tissue repair. Circulating GDF15 levels increase with BMI (6), and a high BMI correlates with poor scar formation after ablation (2), but it remains to be explored whether GDF15 contributes to poor scar formation in a subset of patients with high BMI.

## Funding support

- DGK-Research-Grant of the German Cardiac Society to scholarship recipient (JT).
- Ministry of Science, Research and Cultural Affairs of the State of Brandenburg (CM, WD, KB, BS, OR, GS).

## Supplementary Material

Supplemental data

Supporting data values

## Figures and Tables

**Figure 1:**
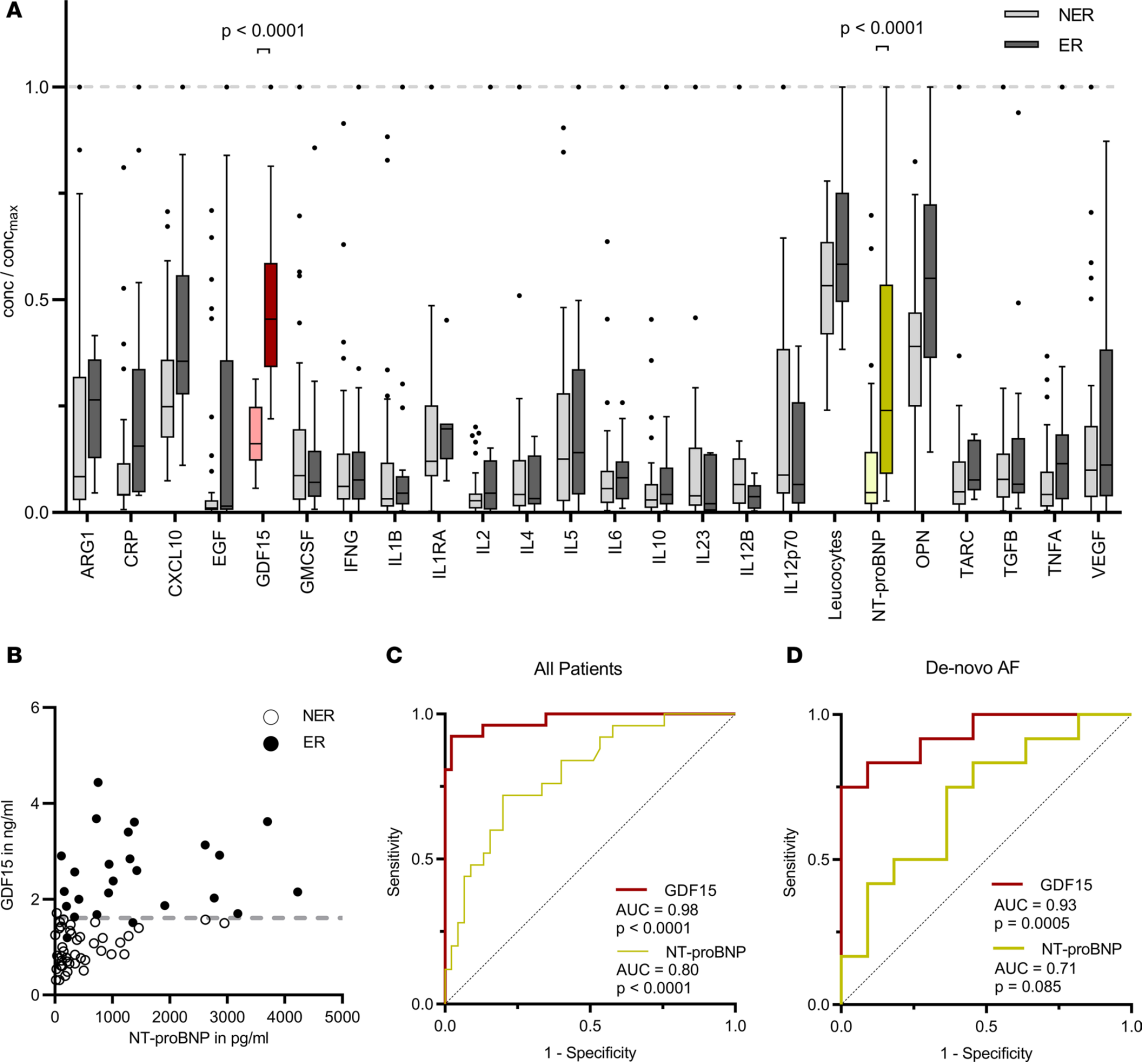
Plasma molecules predicting 24-hour recurrence of atrial arrhythmia after ablation. (**A**) Tukey plots of patient plasma parameters prior to ablation, each normalized to highest value measured. ER, early 24-hour recurrence; NER, no ER. Mann-Whitney *U*/Bonferroni tests were used. (**B**) Scatter comparison of GDF15 and NT-proBNP levels. (**C**) ROC curve analysis of data in **B**. (**D**) ROC curve analysis for de novo patients with AF only.

## Version 1 02/24/2026

In-Press Preview

## Version 2 04/08/2026

Electronic publication
