# Supplementary material for: Plasma GDF15 concentration predicts early recurrence after atrial arrhythmia ablation
Source: JCI Insight. 2026 Feb 24;11(7):e198444. doi: 10.1172/jci.insight.198444 (PMC13134712; doi:10.1172/jci.insight.198444)
Supplement: Supplemental data [file jciinsight-11-198444-s017.pdf]

Supplemental material

Methods details

The study included patients undergoing electrophysiological examination (EP) and catheter ablation for atrial arrhythmia. Participants were 18 years or older, had no cancer or immunosuppressive treatment and were free of signs of infection. This study examined data from male (n = 40) and female (n = 32) individuals. Baseline patient characteristics are outlined in Supplementary Table 1. Treatment during EP was either by RF ablation (n = 48) or cryo ablation (n = 24). Further details on atrial fibrillation patient characteristics and treatment can be found in Table 2.

Table 1. Patient clinical characteristics

	Full cohort (n = 72)	NER (n = 46)	ER (n = 26)	Low GDF15 ≤ 1.61 ng/ml (n = 47)	High GDF15 > 1.61 ng/ml (n = 25)
Age in years, mean ± SD	66.9 ± 13.6	60.5 ± 12.6	76.3 ± 8.4	60.8 ± 12.6	76.4 ± 8.2
Female sex, n (% of group)	32 (45%)	20 (44%)	12 (46%)	22 (47%)	10 (40%)
BMI in kg/m², mean ± SD	29.2 ± 6.2	29.0 ± 5.2	29.4 ± 7.8	28.9 ± 5.1	29.8 ± 7.9
Re-ablation procedure, n (% of group)	25 (35%)	13 (28%)	12 (46%)	13 (28%)	12 (48%)
Atrial Fibrillation, n (% of group)	30 (42%)	15 (33%)	15 (58%)	16 (34%)	14 (56%)
AVNRT, n (% of group)	13 (18%)	11 (24%)	2 (8%)	11 (23%)	2 (8%)
Right ventricular atrial flutter, n (% of group)	8 (11%)	5 (11%)	3 (12%)	5 (11%)	3 (12%)
Left atrial macro re-entry tachycardia, n (% of group)	13 (18%)	8 (17%)	5 (19%)	3 (6%)	5 (20%)
Ectopic atrial tachycardia, n (% of group)	8 (11%)	7 (15%)	1 (4%)	7 (15%)	1 (4%)
HbA1c in %, mean ± SD	5.9 ± 0.8	5.8 ± 0.7	6.3 ± 1.1	5.8 ± 0.6	6.3 ± 1.0
hsCRP in mg/l, mean ± SD	2.0 ± 3.0	1.4 ± 2.2	3.2 ± 3.8	1.9 ± 2.6	3.3 ± 4.0
Leucocytes in 10^9/l, mean ± SD	8.2 ± 2.3	7.5 ± 2.0	9.1 ± 2.5	7.6 ± 2.0	9.0 ± 2.6
NT-proBNP in pg/ml, mean ± SD	829 ± 970	541 ± 647	1514 ± 1270	570 ± 653	1513 ± 1302
EF in %, mean ± SD	58.7 ± 11.3	60.2 ± 9.9	53.4 ± 11.9	60.3 ± 9.7	53.5 ± 11.9
GFR in ml/min, mean ± SD	73.5 ± 22.9	83.4 ± 15.8	56.0 ± 23.5	82.2 ± 16.4	57.1 ± 24.6
CAD, n (% of group)	19 (26%)	10 (22%)	9 (35%)	8 (17%)	11 (44%)

Clinical variables were recorded before EP. NER: non-early recurrence. ER: early recurrence.

Table 2. Atrial Fibrillation patients (n = 30)

Age in years, mean ± SD	70.3 ± 11.5
BMI in kg/m², mean ± SD	28.9 ± 5.5
CHADS VA, mean ± SD	3.4 ± 1.6
paroxysmal AF, n (% of group)	17 (57%)
persistant AF, n (% of group)	13 (43%)
EHRA Score, mean ± SD	2.3 ± 0.7
EF in %, mean ± SD	55.8 ± 8.4
First procedure, n (% of group)	23 (77%)
Re-intervention, n (% of group)	7 (23%)
Antwerpscore, mean ± SD	2.0 ± 1.3
Treatment: Cryo ablation, n (% of group)	24 (80%)
Treatment: RF ablation, n (% of group)	6 (20%)

Clinical variables were recorded before EP.

Blood sample measurement

Plasma concentrations of biomolecules were analysed using FACS-based multiplex ELISA (Legendplex #741397, #740502, BioLegend) or classical ELISA (#DGD150, #DY1433, R&D Systems; #08836752190, Roche). Multiplex assays employed fluorescence–encoded, antibody-coated beads for parallel detection of up to 13 targets. Read-outs were measured using a FACSymphony A3 (BD bioscience), capable of simultaneous detection of up to 30 parameters and 28 colours.

Ablation Procedure and Recurrence Monitoring

Ablation was performed according to current guidelines of the German Society of Cardiology using radiofrequency or cryoablation techniques. All patients were monitored for immediate early recurrence of arrhythmia using a 12 lead ECG, either in response of symptoms within 24 hours post ablation or at 24 hours post ablation before discharge. The primary outcome measure of the study was the rate of arrhythmia recurrence within 24 hours post-ablation.

Heart chamber frequencies

Ventricular and atrial frequencies were measured prior to ablation procedure for linear regression in relation to circulating GDF15 levels. Atrial frequencies from patients with ongoing atrial fibrillation could not be determined. The data shown in figure 1C may therefore underestimate atrial average frequencies of the patient cohort. All patient ventricular frequencies were included (figure 1D).

Sex as a biological variable

Our study examined male and female patients, and similar findings are reported for both sexes.

Statistics

GraphPad Prism Version 9.1 was used for statistical analysis and visualization. Unless noted otherwise, Mann-Whitney-U test was used to test for statistical significance. For post-hoc correction, the significance criterion ($p < 0.05$) was adjusted using the Bonferroni method. Group data were visualized as Tukey's box and whiskers plots. Measurement values given in the text are MEAN±SD.

Binomial logistic regression analysis

To exclude a potential exaggeration of the predictive power of pre-procedure plasma GDF15 concentrations for 24-hours recurrence due to correlation with other factors known to influence arrhythmia recurrence (age, gender, BMI, HbA1c, NT-proBNP, CRP), we performed McFadden binomial logistic regression analysis.

Binomial logistic regression analysis was performed according to McFadden, D. (1974). Conditional logit analysis of qualitative choice behavior. Frontiers in Econometrics P. Zarembka (ed.), 105–142, using formula (1) with an R software package.

(1) $early\ recurrence = age + gender + BMI + HbA1c + NTproBNP + \mathrm{CRP} + \mathrm{GDF15}$

Fitting quality was tested using McFadden's pseudo R^2 (R^2_{McF}). Variance inflation factors (VIF) were calculated to test for collinearity.

Model regression resulted in an exceptionally high $R^2_{McF}=0.78$ with GDF15 as the only significant predictor (Suppl. Table 3). Variance inflation factors were below 2, implying no collinearity problems. Additional testing of all possible models with fewer predictors confirmed this result. Binomial logistic regression with GDF15 alone resulted in $R^2_{McF}=0.74$ (p=0.004).

Table 3. Results of binomial logistic regression

	Effect estimate	Std. Error	z value	Pr(>|z|)
(Intercept)	-17.0260	9.7658	-1.743	0.08
Age	0.0517	0.1036	0.499	0.62
Gender	-0.6655	2.0379	-0.327	0.74
BMI	-0.0350	0.1649	-0.212	0.83
HbA1c	0.0874	0.8104	0.108	0.91
NT-proBNP	-0.0004	0.0008	-0.470	0.64
CRP	0.4149	0.4196	0.989	0.32
**GDF15**	8.3020	4.1367	2.007	**0.04**

Study approval

All clinical investigation has been conducted according to Declaration of Helsinki principles. The study has been approved by the local ethical review board Medical School Brandenburg (Reference number: 161112023-BO-E). A written informed consent was received from participants prior to inclusion in the study.

Data availability

Values for all data points in graphs are reported in the Supporting Data Values file.

Supplementary references on GDF15 and atrial fibrillation recurrence after ablation

Chua W, et al. Disturbed atrial metabolism, shear stress, and cardiac load contribute to atrial fibrillation after ablation: AXAFA biomolecule study. *Europace*. 2024;26(2):euae028.

Fabritz L, et al. Biomarker-based prediction of sinus rhythm in atrial fibrillation patients: the EAST-AFNET 4 biomolecule study. *Eur Heart J*. 2024;45(47):5002-5019.

Gizatulina TP, et al. The soluble ST2 level predicts risk of atrial fibrillation recurrences in long-term period after radiofrequency ablation. *BMC Cardiovasc Disord*. 2024;24(1):460.

El-Harasis MA et al. Recurrence After Atrial Fibrillation Ablation and Investigational Biomarkers of Cardiac Remodeling. *J Am Heart Assoc*. 2024;13(6):e031029.

Supplemental Figure S1.

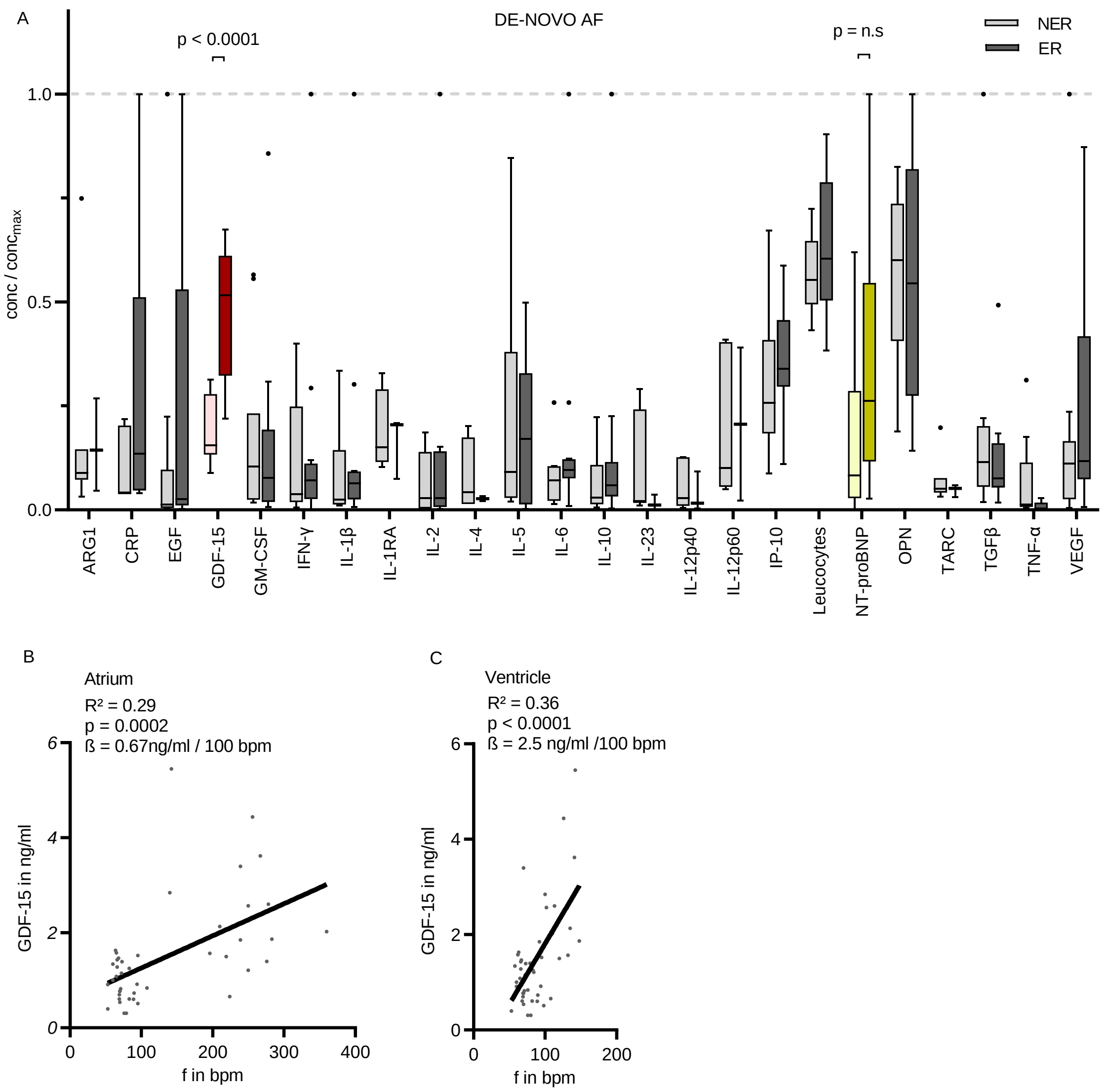

Figure S1: A) Plasma concentrations of serum molecules in de-novo AF patients prior to ablation, each normalized to highest value measured in the total cohort. NER: No early 24-hour recurrence, ER: With early 24-hour recurrence. **(B,C)** Correlation between plasma GDF15 concentrations and atrial **(B)** or ventricular **(C)** heart rate, as measured during procedure after placement of diagnostic catheters.
